# Scale-Up of the Fermentation Process for the Production and Purification of Serratiopeptidase Using Silkworm Pupae as a Substrate

**DOI:** 10.3390/mps7020019

**Published:** 2024-02-25

**Authors:** Jhon Jairo Melchor-Moncada, Alejandra García-Barco, Augusto Zuluaga-Vélez, Luz Angela Veloza, Juan Carlos Sepúlveda-Arias

**Affiliations:** 1 Grupo Infección e Inmunidad, Departamento de Ciencias Básicas, Facultad de Ciencias de la Salud, Universidad Tecnológica de Pereira, Pereira 660003, Colombia; jjmelchor@utp.edu.co (J.J.M.-M.); alejandra.garcia1@utp.edu.co (A.G.-B.); azuluagav@utp.edu.co (A.Z.-V.); 2 Grupo Polifenoles, Facultad de Tecnología, Escuela de Tecnología Química, Universidad Tecnológica de Pereira, Pereira 660003, Colombia; lveloza@utp.edu.co

**Keywords:** serratiopeptidase, fermentation, *Serratia marcescens*, scaling up, purification

## Abstract

Serratiopeptidase, a bacterial metalloprotease known for its pain-relieving and anti-inflammatory properties, can be produced through fermentation with *S. marcescens*. This study aimed to identify key factors related to nutrient composition and physicochemical conditions for production in Erlenmeyer flasks and to scale up the mixture to a bioreactor to obtain the maximum proteolytic activity. A Plackett–Burman design was used to determine whether the presence of silkworm pupae (at 1.5%) was a significant parameter for serratiopeptidase production. Along with the variables pH, temperature, and time, they were optimized using a Taguchi experimental design, resulting in values of 7, 25 °C, and 36 h, respectively. Scaling up with a k_L_a of 25.45 ± 3.12 h^−1^ showed the highest serratiopeptidase production at 24 h. A factorial design was used for ultrafiltration, resulting in an LMH (liters per square meter per hour) of 960 L/m^2^h, a TMP (transmembrane pressure) of 15 psi, and a concentration factor of five, with a specific activity of 24,325.81 ± 1515.69 U/mg. Afterward, the retentate was purified using strong anion exchange chromatography and ultrafiltration, yielding a 19.94 ± 3.07% recovery and a purification factor of 1.59 ± 0.31. In conclusion, waste from the sericulture industry can be used for serratiopeptidase production.

## 1. Introduction

The demand for enzymes is growing significantly worldwide [1]. In particular, hydrolytic enzymes play a significant role in various fields, such as the food and chemical industries, as well as in the biomedical sector [2,3]. One method of enzyme production is submerged fermentation using microorganisms such as bacteria, yeast, or fungi. Although these biomolecules can be obtained from plants and animals, the use of microorganisms is more established [1,4]. Specific conditions are necessary during fermentation to facilitate enzyme production. Some important factors in fermentation include the appropriate nutrients and the physicochemical conditions for achieving the desired outcome [1,5]. The optimization of these factors results in increased fermentation performance and activity of the isolated enzyme as well as a reduction in production costs and the amount of raw material required [6].

One of the strategies for optimizing factors affecting fermentation involves classical optimization methods, such as modifying one factor at a time (OFAT) [5,6]. However, in the conventional approach, assessing the role of each parameter and its influence on product performance is difficult, laborious, slow, and costly [7]. Therefore, statistical methods such as experimental design (DOE) are powerful tools for optimizing fermentation processes [6]. Plackett–Burman factorial designs enable the identification of main factors from a multitude of process variables. Therefore, these designs are useful in preliminary studies where the main goal is to select variables that can be addressed in a subsequent optimization process [8]. When employing this type of statistical experimental design, it is assumed that no interactions occur between different factors within the range of variables under consideration [9]. Among the various optimization tools, the Taguchi method can facilitate the simultaneous optimization of multiple factors and generate quantitative data through fewer experimental trials. This method enhances the reproducibility and efficiency of the process by reducing experimental errors through a set of experimental conditions known as orthogonal arrays, where each phase has a separate yet interconnected objective aimed at achieving the optimization process [7,10]. The successful application of this method for optimizing fermentation process parameters in the production of proteases has been demonstrated [7,11,12].

The commercial production of any microbial product requires production studies at the bioreactor level, as several crucial parameters for cultivation performance, which can be monitored and controlled within the bioreactor, cannot be measured at the flask level. Bioreactor systems are employed for large-scale production to meet industrial product demands via high-performance techniques [1,13,14]. The growing market demand for enzymes drives the need for large-scale production. The scaling-up process in fermentation must preserve integrity and quality while optimizing time to achieve the final product [1]. To scale up the enzyme production process to the bioreactor level, it is essential to maintain a constant volumetric oxygen transfer coefficient (k_L_a) in fermenters of various sizes. The k_L_a value characterizes the efficiency of oxygen transport from the gas phase to the liquid phase in a bioreactor. Factors influencing the k_L_a value include the bioreactor’s geometry, stirrer speed, aeration rate, working volume, and medium properties [15]. Additionally, considerations must be given to nutrient availability, energy expenditure, and the period involved. Therefore, the use of biomass can contribute to the cost-effectiveness of the process [1,14]. Some agro-industrial wastes could serve as alternative sources of carbon and nitrogen during fermentation. These waste products constitute billions of tons of biomass annually and are widely available and renewable [16].

In this context, serratiopeptidase, a protease (hydrolytic enzyme), is obtained from bacteria such as *Serratia marcescens* [17,18,19]. This enzyme is renowned for its anti-inflammatory, fibrinolytic, antibiofilm, and analgesic effects [20,21,22,23,24,25]. The production of serratiopeptidase has been reported using various agro-industrial residues, such as wheat husk and rice bran [7]. Our research group has reported the production of serratiopeptidase using silkworm pupae as a source of carbon and nitrogen [26]. However, the scalability of this fermentation process has not been evaluated. The present study aimed to produce serratiopeptidase using the C8 isolate of *Serratia marcescens* through submerged fermentation. The Plackett-Burman and Taguchi designs were employed, using silkworm pupae as the substrate. Fermentation was scaled up using a bioreactor, with careful consideration of the k_L_a factor. Subsequently, the enzyme was purified using ultrafiltration and chromatographic techniques.

## 2. Materials and Methods

### 2.1. Initial Culture Conditions

The inoculum for fermentation was obtained by cultivating the C8 isolate of *Serratia marcescens* on nutrient agar at 26 °C for 18 h. Subsequently, a 0.5 McFarland standard solution was prepared and added to the culture medium at a final concentration of 1%. Fermentation occurred in a 250 mL Erlenmeyer flask without baffles, which was filled to 20% capacity (50 mL) and agitated at 180 rpm.

The silkworm pupae used as a source of nutrients for fermentation were obtained from the El Pilamo experimental farm located in Risaralda, Colombia. This material was dried at 50 °C for 2 days. The samples were ground for analysis, and the moisture content was determined by weighing the samples at 105 °C for 24 h. On the other hand, fats and oils were extracted using the Soxhlet method, and ashes were obtained by calcination in a muffle furnace at 600 °C for 24 h following AOAC standards. Finally, crude protein was determined using the Kjeldahl method with a factor of 6.25, and carbohydrates and other factors were calculated by multiplying the difference from the obtained results by 100% (Figure S1).

### 2.2. Proteolytic Activity and Total Protein Assay

The protocol used to determine proteolytic activity was described by Vélez-Gómez et al. Briefly, azocasein was used to assess the proteolytic activity of the sample, which was incubated at 37 °C for 10 min. A calibration curve of the azopeptides was then generated (see Figure S2) and measured at 440 nm. Proteolytic activity was determined according to Equation (1).

(1) $$\mathrm{Proteolytic}\;\mathrm{activity}=\frac{Azopeptides\;\left(\mu g\right)\;*\;Reaction\;volume\;\left(mL\right),}{Incubation\;time\;\left(min\right)*Sample\;volume\;\left(mL\right)*Aliquot\;\left(mL\right)},$$

The quantification of total soluble protein was conducted using the Bradford assay, and the results were compared with a calibration curve derived from bovine serum albumin (BSA), as illustrated in Figure S3.

### 2.3. Plackett-Burman and Taguchi Designs

The determination of significant nutrients during the process was carried out at the agitation flask level through a Plackett–Burman experimental design to identify significant components and to determine the optimal combination of nutrients for the production of serratiopeptidase, focusing on proteolytic activity [27]. Twelve experiments were conducted to assess the relative importance of six variables, namely, silkworm pupae, casein, soybean oil, ammonium dihydrogen phosphate ((NH_4_)_2_HPO_4_), zinc chloride (ZnCl_2_), and calcium chloride (CaCl_2_·2H_2_O). For each variable, both high and low concentrations were employed (Table 1). Protease activity was considered to indicate an experimental response at a significance level of 0.05, with the uninoculated ferment used as the blank. Minitab Statistical Software Version 21.2 was used for the analysis of the experimental design, and measurements were taken in a random order.

The fermentation process was optimized at the agitation flask level through a Taguchi experimental design to achieve the optimal combination of conditions for serratiopeptidase enzyme production [7]. The study evaluated the relative importance of four variables, namely, silkworm pupa abundance, temperature, time, and pH. Each factor was represented at three levels, except for the silkworm pupae, which had six levels (Table 2).

The prediction and confirmation of the Taguchi experimental design aim to bring the average response closer to the target value [28]. Following the determination of optimal parameters through the Taguchi experimental design, the response variable (proteolytic activity) was predicted using these optimal parameters to confirm the design.

### 2.4. The Fermentation Process Was Scaled Up

A 5-L bioreactor (Bioengineering, Figure S4) was used with the static gasification method to determine the oxygen transfer coefficient (k_L_a) for scaling up the fermentation process. Agitation and dissolved oxygen conditions were assessed by varying the stirring speed and aeration rate, and tests were conducted in triplicate. Initially, the oxygen concentration was reduced to values close to zero by passing gaseous nitrogen through the system. After suspending the nitrogen flow, aeration commenced under established operating conditions, including the stirring speed and aeration rate. Dissolved oxygen levels were measured using an electrode. Notably, in the experimental bioreactor, no viable cells were present [29]. The experiments were performed under optimized medium conditions. To determine k_L_a values, evaluations were carried out at 100, 200, and 300 rpm in combination with 0.5, 1.0, and 1.5 vvm (volume of air per volume of medium per minute).

### 2.5. Optimization of Enzyme Purification

The product of the scaling process was centrifuged at 15,500× *g* for 15 min. The resulting supernatant was then filtered through a 0.45 µm PVDF membrane, followed by a 0.22 µm membrane. Subsequently, a tangential flow filtration process (ultrafiltration) was employed using a 10 K cartridge. This process serves the dual purpose of concentrating the crude extract, reducing the working volume, and decreasing the concentration of salts and molecules with a molecular weight lower than the membrane cutoff size. The ultrafiltration process was optimized utilizing Pall Minimate equipment and a factorial design. The experiment was conducted with various transmembrane pressure (TMP) values (10, 15, and 25 psi) and process feed fluxes (LMHs) (360, 960, and 1560 L/m^2^h) at a concentration factor of 5. Then, diafiltration was performed at a flux rate of 960 L/m^2^h and 15 psi using Tris-HCl (25 mM) and CaCl_2_·2H_2_O (1 mM) buffer at pH 7.

The concentrated and diafiltrated supernatant was purified using fast protein liquid chromatography (FPLC) with a UNOsphere Q anion exchange column (Bio-Rad, Hercules, CA, USA) on a Biologic DuoFlow 10 system (Bio-Rad). The purification involved a single-step process in which the column was preequilibrated with buffer A (25 mM Tris-HCl + 1 mM CaCl_2_, pH 7) and eluted at 3 mL/min with buffer B (25 mM Tris-HCl + 1 mM CaCl_2_ + 1 M NaCl, pH 7). The elution gradient was as follows: 0–15% B, 5 CV; 50% B, 2 CV; 100% B, 1 CV; 0% B, 2 CV (the remaining percentage corresponds to buffer A). Proteolytic activity and protein levels were monitored as response variables in all patients.

The kinetics of fermentation, chromatographic purification, and purification of the enzymes were monitored using a size exclusion molecular column (EnRich SEC-70, Bio-Rad; flow rate, 1 mL/min; mobile phase, Tris-HCl 25 mM + 1 mM CaCl_2_; pH 8). Molecular weights were determined by SEC utilizing a calibration curve (bovine thyroglobulin 670 kDa, bovine γ-globulin 158 kDa, chicken ovalbumin 44 kDa, equine myoglobin 17 kDa, and vitamin B12 1.35 kDa; Figure S5). The enzyme was also monitored using SDS-PAGE electrophoresis with a 15% separation gel run at 100 V. The electrophoresis process was carried out using 50 μg of each sample (concentration determined using the Bradford method). Proteins were stained with 0.25% Coomassie R-250 Brilliant Blue in a mixture of methanol–acetic acid–water (5:1:4). The gel was subsequently decolorized with a methanol–acetic acid–water mixture (5:1:4). The Thermo Scientific Broad Range Protein Ladder (10–260 kDa) and Abcam Prism Protein Ladder (10.5–175 kDa) served as molecular weight standards (see curves in Figure S6a,b).

## 3. Results

### 3.1. Plackett–Burman Design

A Plackett–Burman experimental design was used to assess the effects of six nutrients (silkworm pupae, casein, soybean oil, (NH_4_)_2_HPO_4_, ZnCl_2_, and CaCl_2_·2H_2_O) in fermentation media, aiming to determine the optimal nutrient combination for serratiopeptidase production at the agitation flask scale. These nutrients, which were identified as significant factors influencing serratiopeptidase production, were based on previous research [26]. The results of the experimental design, expressed in terms of proteolytic activity in U/mL, are presented in Table S1.

**Figure 1:**
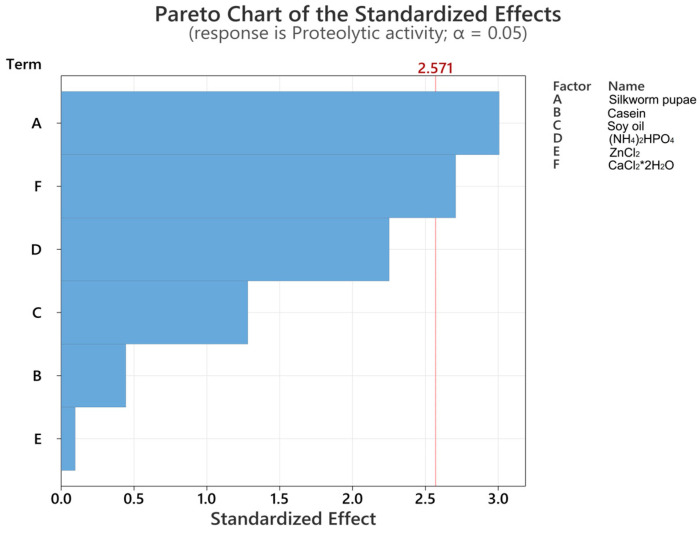
Pareto plots for proteolytic activity.

In Figure 1, the Pareto diagram reveals the statistically significant variables in the study. Figure 1 revealed that silkworm pupae were the most impactful variable, making a positive contribution to serratiopeptidase production (*p* = 0.030). This difference is likely attributable to the substantial content of protein (47.25%), carbohydrates (28.00%), and fats and oils (13.50%) found in the silkworm pupae (refer to Figure S1). These components can potentially serve as valuable sources of carbon and nitrogen in the serratiopeptidase production process.

The presented model exhibited a strong correlation of 82.33%, as indicated by Equation (2), suggesting that the model was fit with a biological model [30]. This correlation underscores the robust relationship between the studied variables and supports the notion that silkworm pupae play a crucial role in enhancing serratiopeptidase production within the experimental framework.

Proteolytic activity = 724 + 602 Silkworm pupae + 93 Casein + 338 soy oil − 751 (NH_4_)_2_HPO_4_ − 253 ZnCl_2_ + 7134 CaCl_2_·2H_2_O, (2)

The positive contribution of silkworm pupae (coefficient 602) is evident in Equation (2), signifying that an increase in this variable is correlated with an increase in proteolytic activity. Conversely, calcium chloride, the second most significant variable, with a *p* value of 0.042 (Figure 1), has a positive contribution, with a coefficient of 7134. These findings suggest that a higher concentration of calcium can enhance the production of serratiopeptidase, given that this enzyme requires seven calcium cofactors [31]. Consequently, the upper limit concentration for this variable was used to advance the optimization process. For variables such as zinc chloride and ammonium dihydrogen phosphate, which exhibited a negative contribution, lower concentration limits were employed. Casein and soybean oil were applied at the lower limits to incorporate statistically significant variables. In other words, the residue from the sericulture industry is utilized as a source of carbon and nitrogen. Maintaining a concentration of casein in the culture medium is crucial, as it has been demonstrated to act as an inducer of the formation of serratiopeptidase by *Serratia marcescens* [32].

### 3.2. Taguchi Design

The most significant variable, in this case, silkworm pupae, as determined by the screening experimental design (Plackett-Burman), was incorporated into the optimization process along with other physicochemical variables that proved to be significant, including pH, temperature, and fermentation time.

A matrix L18 with 18 different experiments was constructed based on the experimental data obtained in the Plackett–Burman experimental design and in preliminary experiments [26]. Table S2 displays the Taguchi design utilized and the response in terms of the proteolytic activity.

**Figure 2:**
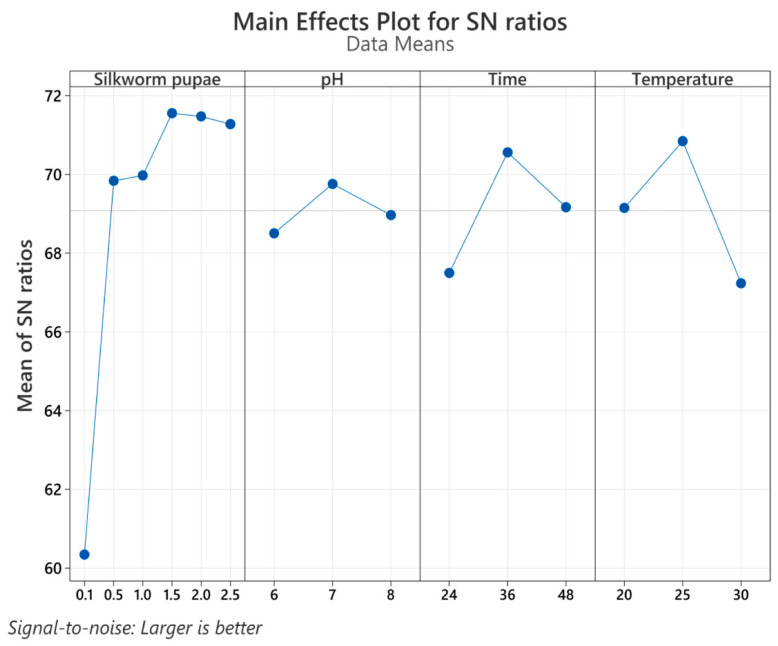
Main effects plot for S/N ratios. The results are presented as the means ± standard deviations of three independent experiments.

Figure 2 presents the results of the main effects for signal-to-noise (S/N) ratios, assuming “larger is better”. This Taguchi analysis identified key factors for achieving maximum serratiopeptidase production. Figure 2 shows that the most influential parameters are 1.5% *w*/*v* silkworm pupae, a pH of 7, a fermentation time of 36 h, and a fermentation temperature of 25 °C. Notably, compared with the other variables, silkworm pupae exhibited a significant contribution (*p* = 0.003), as shown in Table S3. Employing the variable values for achieving maximum serratiopeptidase production, the model predicted a value of 4724.07 U/mL. Compared with the experimental values obtained under model validation conditions, an experimental proteolytic activity value of 4501.23 ± 398.21 U/mL was obtained. The results demonstrated high agreement between the predictive and experimental outcomes, with an error percentage of less than 5%. Furthermore, the model exhibited a strong correlation of 93.34%, confirming its precision.

Proteolytic activity = 1222 + 917 Silkworm pupae + 157 pH + 32.0 Time − 59.0 Temperature,(3)

In accordance with Equation (3), which was derived from the Taguchi experimental design model, the variables exerting the most significant influence on proteolytic activity were the number of silkworm pupae and the pH, both of which exhibited the highest positive coefficients. Time also positively contributed to proteolytic activity, albeit to a lesser extent (a coefficient of 32), whereas temperature exerted a negative influence on proteolytic activity. This contradicts the dynamics of serratiopeptidase production, where elevated temperatures adversely impact *Serratia marcescens* bacteria, leading to a diminished enzyme yield [7].

### 3.3. The Fermentation Process Was Scaled Up

The scaling-up conditions included 1.5% silkworm pupae, 0.1% casein, 0.1% soybean oil, 0.5% ammonium dihydrogen phosphate, 0.01% zinc chloride, 0.2% calcium chloride, 1% inoculum at 0.5 McFarland, pH 7, and a temperature of 25 °C. The agitation speed and airflow were adjusted to match a k_L_a value similar to that calculated at the laboratory flask level (23.23 h^−1^) [33]. Additionally, the fermentation time was reassessed under bioreactor conditions.

#### 3.3.1. Determination of the Volumetric Oxygen Transfer Coefficient (k_L_a)

The optimized culture medium was deoxygenated using nitrogen, and the agitation speed and air volume per volume of medium per minute (vvm) were tested in combination, as described in Table S4. Dissolved oxygen (DO) levels were monitored in all patients. To convert vvm to air flow in standard liters per hour (SL/h), Equation (4) was used, where the working volume (V_w_) was 2.7 L.

Air flow (SL/h) = vvm × V_w_ × 60, (4)

**Figure 3:**
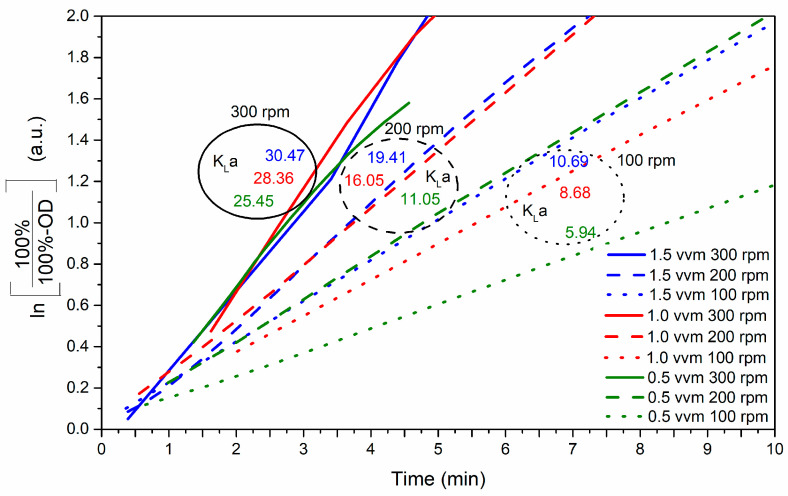
The volumetric oxygen transfer coefficient (k_L_a) was obtained for different vessel volumes per minute (vvm) and agitation (rpm). The results represent the average of three independently conducted trials.

For each experiment, dissolved oxygen (DO) was converted to the natural logarithm (Ln(100%/(100%-DO))) and plotted over time. The slope of the linear segment represents the k_L_a value, as illustrated in Figure 3. The k_L_a values in the fermenter were sensitive to an increase in agitation speed. A coefficient of 10.69 h^−1^ was achieved at 100 rpm and 1.5 vvm. Extrapolating these findings implies that attaining a k_L_a value comparable to that of an agitated flask necessitates a higher air flow rate. However, this approach is economically impractical due to the associated air consumption. Conversely, an excessive agitation speed could have detrimental effects on the viability of bacteria during fermentation [34].

In particular, k_L_a values ranging from 25.45 h^−1^ to 30.47 h^−1^ were achieved at an agitation speed of 300 rpm, coupled with various aeration rates. These results underscore the pivotal role of the input energy (rpm) in increasing the gas-liquid oxygen mass transfer rate. Consequently, 300 rpm and 0.5 vvm in the bioreactor (25.45 h^−1^) were identified as conducive to attaining a volumetric oxygen transfer coefficient, k_L_a, comparable to that of the agitated flask (23.23 h^−1^). Subsequently, fermentation was scaled up under these optimized conditions, integrating values of 0.5 vvm and 300 rpm.

#### 3.3.2. Kinetics of Serratiopeptidase Production at the Bioreactor Level

Fermentation conditions were methodically controlled to validate the production of serratiopeptidase, with assessments conducted at both the Erlenmeyer flask and 5 L bioreactor scales. Kinetic analysis was employed in the bioreactor to determine the optimal production time for serratiopeptidase in terms of the proteolytic activity.

**Figure 4:**
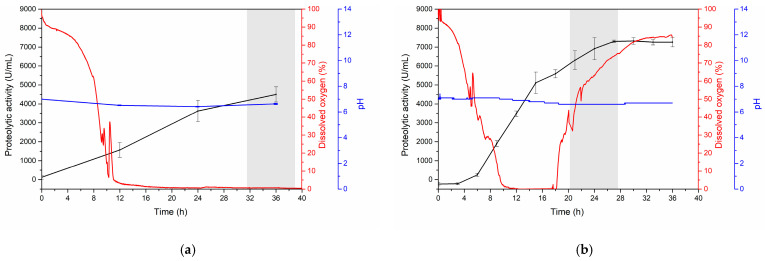
Kinetics of proteolytic activity production in (**a**) an Erlenmeyer flask and (**b**) a 5 L fermenter with the C8 isolate using optimized conditions for silkworm pupae, pH, and temperature. The data are presented as the mean ± SD of three independent experiments.

Figure 4a,b display the monitoring of the dissolved oxygen, pH, and proteolytic activity of both fermentations. The figures reveal a notable difference in terms of the proteolytic activity. In the Erlenmeyer flask fermentation, a peak production of approximately 4500 U/mL was observed at 36 h, while in the bioreactor, it reached approximately 6800 U/mL at approximately 24 h. Therefore, scaling up is inferred to result in higher efficiency (Figure 4a,b). This observation may be attributed to the dissolved oxygen curve, where the initial 16 h exhibited similar behavior in both fermentations. However, bioreactors benefit from a constant air supply, contributing to the observed difference.

Analyzing the activity curve (Figure 4b), a diauxic behavior is evident at 16 h, reaching a value of approximately 5000 U/mL. This phenomenon could be associated with the consumption of secondary nutrients combined with a continuous air supply stimulating cell growth and, consequently, enhancing proteolytic activity [34,35]. Notably, the difference in pH between the two fermentations was not statistically significant.

**Figure 5:**
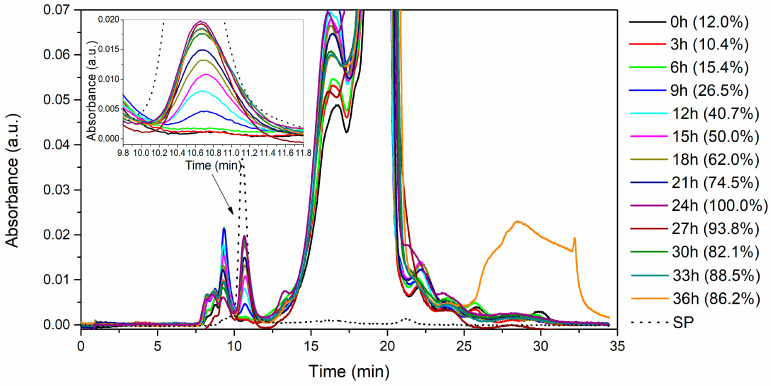
Size exclusion chromatography (SEC) was used to monitor the fermentation process in a 5-L bioreactor using a silkworm pupae substrate.

**Figure 6:**
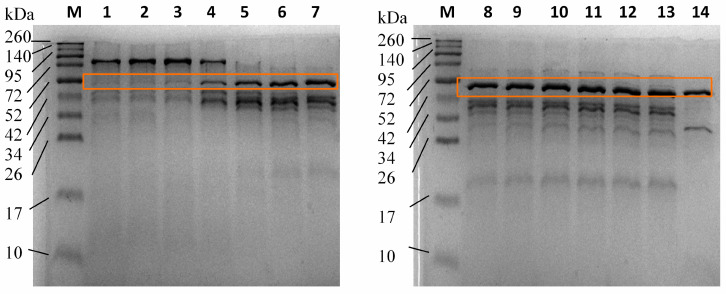
SDS-PAGE was used to monitor the fermentation process in a 5-L bioreactor using a silkworm pupae substrate. M: molecular ladder, 1: 0 h, 2: 3 h, 3: 6 h, 4: 9 h, 5: 12 h, 6: 15 h, 7: 18 h, 8: 21 h, 9: 24 h, 10: 27 h, 11: 30 h, 12: 33 h, 13: 36 h, and 14: serratiopeptidase. The orange box indicates the molecular weight corresponding to serratiopeptidase.

An increase in proteolytic activity was associated with the production of the serratiopeptidase enzyme, as confirmed by monitoring through size-exclusion chromatography (SEC) and SDS-PAGE (Figure 5 and Figure 6). Pure serratiopeptidase (SP) served as the benchmark for comparison.

In Figure 5, the distinct peak at 10.6 min corresponds to pure serratiopeptidase. By quantifying the area under the curve associated with this enzyme peak for each fermentation time (Figure 5, on the right within parentheses), a correlation was established with the kinetics presented in Figure 4b. Notably, the 24 h fermentation period exhibited the highest enzyme quantity based on the area under the curve. Furthermore, the elution volume of the 10.6 min peak facilitated the determination of the enzyme’s molecular weight, which was subsequently compared with the standard calibration curve (refer to Figure S5). The resulting molecular weight was determined to be 51.35 ± 0.67 kDa.

These results (Figure 6) align with the proteolytic activity profile depicted in Figure 4b. Figure 6 reveals a minimal band at approximately 50 kDa associated with the serratiopeptidase enzyme during the initial hours of fermentation (lanes 2 and 3). As time progresses, enzyme production becomes apparent from 9 h onward (lane 4), leading to a gradual intensification of the 50 kDa band. The molecular weight, determined through this analytical method, was calculated as 50.35 ± 0.11 kDa. This value was derived using a calibration curve with a molecular weight marker (see Figure S6a).

### 3.4. Enzyme Purification

**Figure 7:**
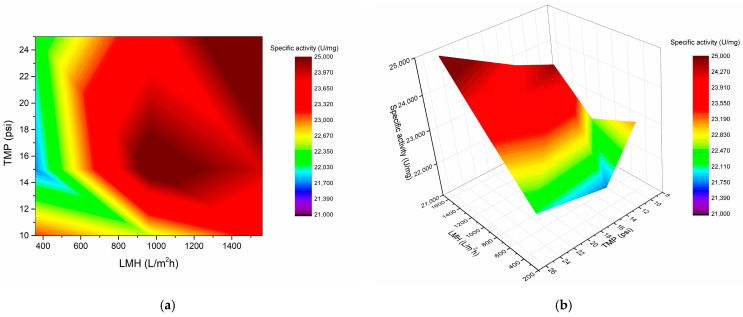
Factorial design, TMP and LMH variables: (**a**) surface color map and (**b**) 3D surface for the ultrafiltration process.

The product obtained from a 24 h fermentation under scaled-up conditions underwent an initial step of ultrafiltration. To achieve this goal, the centrifuged and filtered supernatants were analyzed using a two-variable factorial design. The independent variables included transmembrane pressure (TMP) and flux (LMH), with specific activity serving as the response variable. The specific activity considered both the proteolytic activity and total protein quantity. It is important to note that membrane permeability was consistently monitored and cleaned between each trial as per the supplier’s instructions. A total of nine trials were conducted in triplicate, using 250 mL aliquots and varying TMPs from 10 to 25 psi and LMHs from 360 to 1560 L/m^2^h. A 10 kDa molecular weight cutoff (MWCO) was used for the ultrafiltration process. The results are illustrated in Figure 7a,b. The optimal conditions for TMP and LMH were identified at 25 psi and 1560 L/m^2^h, resulting in a specific activity of 25,016.23 ± 1359.88 U/mg. Additionally, conditions at 15 psi and 960 L/m^2^h yielded a specific activity of 24,325.81 ± 1515.69 U/mg (areas of higher color intensity in Figure 7a,b). Both results showed no significant differences. Consequently, the purification process was carried out under lower pressure and flux conditions to preserve the longevity of the ultrafiltration cartridge.

After concentrating the samples through ultrafiltration and determining the transmembrane pressure and process flow, the continuous or constant-volume diafiltration technique is employed to wash out salts and low-molecular-weight species present in the concentrated (retained) sample. An LMH of 960 L/m^2^h and a pressure of 15 psi were used, and one volume of diafiltration was performed with Tris-HCl 25 mM buffer, CaCl_2_·2H_2_O, 1 mM, pH 7.

**Figure 8:**
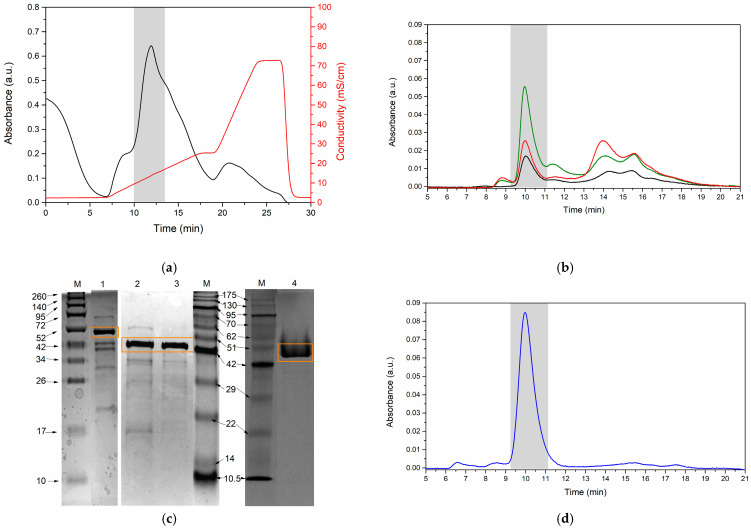
Optimization of enzyme purification. (**a**) Strong anion-exchange chromatographic (IEX) profiles of ultrafiltration samples of silkworm pupae at 280 nm. The active fraction is shown in gray shadow; (**b**) chromatographic profiles from anion-exchange fractions. The active fraction is shown in gray shadow; (**c**) monitoring of purification steps by SDS-PAGE, M: molecular ladder, 1: crude extract, 2: diafiltration TFF 10 kDa, 3: IEX, 4: purified enzyme, and (**d**) chromatogram by SEC of the purified enzyme (95.09% purity). The orange box indicates the molecular weight corresponding to serratiopeptidase.

The filtered product was purified via strong anion-exchange chromatography (IEX) and was monitored at 280 nm (Figure 8a). The chromatogram exhibits a shaded region at approximately 11 min, indicating fractions with proteolytic activity. The presence of serratiopeptidase in the IEX-collected fractions was confirmed through size-exclusion chromatography (SEC), as shown in Figure 8b. The major peak corresponds to the retention time associated with serratiopeptidase; the detail was previously elucidated Figure 5. Despite this, the presence of lower-molecular-weight impurities was noticeable, necessitating further refinement steps in the purification process. Subsequently, the IEX-derived fractions were concentrated and diafiltrated using Macrosep (Pall). This step aimed to increase the purity of the serratiopeptidase by minimizing impurities with lower molecular weights. The entire purification process involved SDS-PAGE (Figure 8c) and a calibration curve (Figure S6b), which revealed the presence of a serratiopeptidase-associated band (50.25 ± 0.137 kDa). Finally, SEC was employed to monitor the purified enzyme. Figure 8d shows the chromatogram corresponding to the serratiopeptidase, displaying an area under the curve of 95.09%. This result underscores the efficacy of the purification strategy employed and the successful isolation of serratiopeptidase with a high degree of purity. The purification results are outlined in Table 3 and encompass the total protein content, specific activity, total proteolytic activity, yield, and purification factor.

At the beginning of fermentation, a protein content of 1762.50 mg was expected, which equated to 47% of the content of the silkworm pupae used in the process. However, protein quantification using the Bradford assay revealed a value of 70.09 mg (Table 3), indicating a significant discrepancy. This difference can be explained by the results obtained from size-exclusion chromatography (Figure 5), which indicate that the culture medium containing silkworm pupae contained an abundance of proteins and peptides of low molecular weight (peaks observed between 15 and 20 min with higher intensity). This observation is supported by a study conducted by Li et al. [36], who identified a peptide with the sequence PNPNTN corresponding to the low-molecular-weight fraction. In particular, the reduced presence of basic amino acids in these biomolecules suggested that the Bradford method may not accurately quantify the total amount of proteins and peptides during the purification process. Therefore, when performing size-exclusion chromatography at 280 nm (see Figure 8b) for protein monitoring during the purification steps, the loss of low-molecular-weight biomolecules was evident. Consequently, the quantity reported by the Bradford assay showed no significant changes between the purification steps, resulting in a purification factor of only 1.59. However, the purification process was supported by size-exclusion chromatography, wherein a clear reduction in low-molecular-weight impurities was observed.

The initial crude extract had an activity of 6446.91 U/mL, equivalent to 1,611,728.39 U for the 250 mL aliquot. The yield and purification factor were 100% and one, respectively [37]. In the second step of ultrafiltration, the purification factor was 1.06, and the sample was concentrated 5-fold with a 96.60% recovery. This indicates that tangential ultrafiltration through a 10 kDa membrane results in the highest amount of proteolytic activity being retained in a smaller volume. In this step, the protein content (63.79 mg) decreased, increasing the specific activity to 24,325.81 U/mg. This third step of purification showed a 57.62% reduction in recovery and an increase in the purification factor to 1.21. The diafiltrated sample was then subjected to IEX, obtaining recovery and purification parameters similar to those of the previous step (54.42% and 1.19%). In Table 3, a protein loss of 1.39 mg and a total activity loss of 54,037.03 U were observed. This step allowed for the removal of impurities present in the sample. Finally, the purification was refined using ultrafiltration through Macrosep, which achieved a yield of 19.94%, a purification factor of 1.59, and a specific activity of 36,152.12 U/mg with a purity of 95.06% (Table 3 and Figure 8d).

## 4. Discussion

Serratiopeptidase, known for its fibrinolytic and anti-inflammatory properties, shows promise for a range of biomedical applications [38,39,40,41]. However, its susceptibility to pH and temperature fluctuations necessitates efficient production and purification methods. Through the Plackett–Burman factorial design, silkworm pupae were identified (with a *p* value < 0.05) as a potential substrate for serratiopeptidase production from the C8 isolate of *Serratia marcescens* (Figure 1). As reported by Yeruva et al., silkworm pupae exhibit a substantial protein content ranging between 51 and 55% and are rich in essential amino acids [42]. Hence, our findings suggest that silkworm pupae offer an alternative source of carbon and nitrogen suitable for use in the fermentation process [26,43].

Several previous studies have indicated that the use of agro-industrial residues can enhance the production of proteases [31,44,45,46,47]. Additionally, enzyme production can benefit from the presence of nutritional sources and the overall growth and metabolism of the microbial strain [4,5,14]. Moreover, the optimization of significant physicochemical variables such as pH, temperature, and fermentation time through the Taguchi methodology demonstrated a strong preference for serratiopeptidase production at pH 7, a temperature of 25 °C, an incubation time of 36 h, and a silkworm pupa concentration of 1.5% (shown in Figure 2). It is plausible that the maximum proteolytic activity observed during this fermentation period in Erlenmeyer flasks could be attributed to the availability of primary nutrients and dissolved oxygen (Figure 4a), as indicated by several studies [29,34,44,47].

Oxygen plays a crucial role in the production of serratiopeptidase from *Serratia marcescens*, as this bacterium relies heavily on oxygen to synthesize specific metabolites [34]. Previous studies have indicated that proteases can be generated from agro-industrial residues through fermentation with *S. marcescens* [7,48]. Temperature is also a critical parameter in this process, as it plays a significant role in microbial growth and product formation. Studying the impact of different temperatures (20, 25, and 30 °C) revealed that the maximum enzyme production was obtained at 25 °C. These results align with findings reported by Pansuriya et al. and Fahmy et al., both of whom observed maximum protease production at 25 °C [47,49]. The hydrogen ion concentration promotes microbial growth and product formation by regulating the transport of various metabolites and nutrients across the cell membrane [50]. Evaluating the effect of different pH values (6, 7, and 8) on serratiopeptidase production revealed that the optimal pH for maximum enzyme production is pH 7, with the lowest yield occurring at pH 6. Bach et al. reported similar results when producing a protease from *Serratia marcescens* P3, investigating the influence of pH on enzyme production and finding maximum production at pH values between 6.5 and 8.5 [51].

Based on the results obtained, the fermentation process was scaled using k_L_a. Figure 3 shows that dissolved oxygen is strongly influenced by agitation. The conditions used for the scaling process were 0.5 vvm and 300 rpm. Overall, this study contributes insights into the critical parameters affecting k_L_a, which is consistent with prior research emphasizing the importance of optimizing these parameters for efficient and scalable bioprocesses [34,35]. This comprehensive approach not only ensures the optimization of the culture medium but also provides a robust framework for the precise control and measurement of key variables, ultimately contributing to the reproducibility and reliability of the experimental outcomes [52]. Figure 4b illustrates the effect of oxygen on the scaled-up process, which is more efficient than the other processes and results in more proteolytic activity (6800 U/mL) in less time (24 h). Additionally, a diauxic phenomenon is observed, wherein the presence of oxygen, fluctuations in temperature or pH, and the use of complex nutrients collectively contribute to the observed effects [34,35,53].

The observed increase in proteolytic activity was directly attributed to the synthesis and secretion of the serratiopeptidase enzyme. This relationship was validated through meticulous monitoring involving size-exclusion chromatography and SDS-PAGE analyses, as depicted in Figure 5 and Figure 6. Size-exclusion chromatography enables the separation of molecular components based on their size, providing a detailed profile of the enzymatic fractions present in the fermentation broth. Concurrently, SDS-PAGE, a widely utilized electrophoretic technique, further validated the identity and purity of the serratiopeptidase, ensuring that the detected activity indeed stemmed from the target enzyme. The combined application of these analytical techniques not only confirmed the association between increased proteolytic activity and serratiopeptidase production but also provided valuable insights into the structural integrity and purity of the enzymes, which are essential aspects for further downstream applications and industrial-scale processes. Notably, these results are consistent with reported findings on various metalloproteases isolated from distinct strains of *S. marcescens*, which have molecular weights within the range of 43 to 60 kDa [26,54,55,56,57,58,59].

Optimization and upscaling of the conditions for serratiopeptidase production have been successfully achieved. However, attention must be directed toward efficient purification methods. While the use of ultrafiltration for similar fermentations has been documented [26], the specific conditions impacting the ultrafiltration process have yet to be assessed. Thus, as illustrated in Figure 7, 960 L/m^2^h and 15 psi resulted in the highest levels of proteolytic activity. This approach demonstrates greater efficiency than do conventional methods such as precipitation with ammonium sulfate [51,60].

Finally, the enzymatic purification steps are summarized in Figure 8 and Table 3, which offer a comprehensive overview of the process. Notably, a yield of 54.42% was achieved during the chromatography step, surpassing the yields reported by previous studies conducted by Vélez et al. [26] and Nageswara et al. [60], whose yields were 32.27% and 34%, respectively. It is worth mentioning that while our yield exceeded these reported values, it fell slightly below the yield reported by Srimathi and Virivinti [46]. This discrepancy in yields could be attributed to variations in experimental conditions, such as differences in purification techniques, optimization parameters, or variations in the characteristics of the enzyme source. Additionally, it is important to note that while our yield is lower than that in the study by Srimathi and Virivinti [46], our purification process still represents a significant improvement over previously reported methods, highlighting the effectiveness of our approach in achieving a high level of enzyme purity.

Based on these findings, silkworm pupae may be a potential source of carbon and nitrogen for the C8 isolate of *S. marcescens*. While this approach is promising, further enhancements using high-resolution columns are necessary to achieve nearly 100% purity. Notably, preliminary toxicity assessments are crucial, especially for assessing biological activities such as fibrinolysis and inflammation [38]. Silkworm pupae have emerged as promising substrates for serratiopeptidase production, suggesting avenues for further exploration in both research and industrial applications. Our study underscores the importance of optimizing both production and purification processes to advance the development of efficient enzyme-based therapies.

## 5. Conclusions

The use of silkworm pupae in fermentation media and the scaling up of the fermentation process for the production of serratiopeptidase increased the efficiency of production. Moreover, this method establishes a fermentation process based on the principles of the circular economy, which adds value to the sericulture industry. Furthermore, it was possible to purify the serratiopeptidase enzyme obtained under scaled-up conditions using ultrafiltration and a single chromatographic step.

## Figures and Tables

**Table 1:** Plackett–Burman design for determining significant nutrients for serratiopeptidase production.

Factor	Silkworm Pupae (%)	Casein (%)	Soy Oil (%)	(NH_4_)_2_HPO_4_ (%)	ZnCl_2_ (%)	CaCl_2_·2H_2_O (%)
High	2.50	2.50	2.00	2.00	0.20	0.20
Low	0.00	0.10	0.10	0.50	0.01	0.01

The percentage refers to the ratio of mass to volume.

**Table 2:** Taguchi design. Mixed-level design. L18 (6^1^), (3^3^).

Trial Number *	pH	Temperature (°C)	Time (h)	Silkworm Pupae (%*^w^*^/*v*^)
1	6	20	24	0.1
2	7	25	36	0.1
3	8	30	48	0.1
4	6	25	24	0.5
5	7	30	36	0.5
6	8	20	48	0.5
7	6	20	36	1.0
8	7	25	48	1.0
9	8	30	24	1.0
10	6	30	48	1.5
11	7	20	24	1.5
12	8	25	36	1.5
13	6	30	36	2.0
14	7	20	48	2.0
15	8	25	24	2.0
16	6	25	48	2.5
17	7	30	24	2.5
18	8	20	36	2.5

* The experiments were carried out in a random order.

**Table 3:** Purification steps of serratiopeptidase produced by fermentation using silkworm pupae.

Sample	Protein (mg)	Specific Activity (U/mg)	Total Activity (U)	Recovery (%)	Purification Fold
Crude extract	70.09 ± 2.77	22,983.32 ± 1561.35	1,611,728.39 ± 142,346.87	100.00	1.00
Ultrafiltration TFF 10 kDa	63.79 ± 1.53	24,325.81 ± 1515.69	1,550,370.37 ± 67,709.37	96.60 ± 8.15	1.06 ± 0.07
Diafiltration TFF 10 kDa	33.33 ± 1.30	27,758.61 ± 879.48	924,333.33 ± 6555.55	57.62 ± 4.50	1.21 ± 0.11
Strong anion exchange	31.94 ± 0.61	27,269.88 ± 1845.97	870,296.30 ± 44,954.06	54.42 ± 7.21	1.19 ± 0.15
Ultrafiltration 10 kDa	8.86 ± 0.61	36,152.12 ± 1708.81	318,474.07 ± 23,735.51	19.94 ± 3.07	1.59 ± 0.31

## Data Availability

The data presented in this study are available on request from the corresponding author.

## Supplementary Materials

The following supporting information can be downloaded from https://www.mdpi.com/article/10.3390/mps7020019/s1, Figure S1: Proximate analysis of silkworm pupae; Figure S2: Calibration curve for determination of azopeptide amounts using azocasein; Figure S3: Calibration curve for determination of total protein by Bradford assay; Figure S4: Scaling up in a Bioengineering RALF Advanced Bioreactor; Figure S5: Calibration curve for determination of relative molecular mass. As standards, γ-globulin (bovine), albumin (chicken), myoglobulin (horse) and vitamin B12 were used. Figure S6: SDS-PAGE molecular weight marker calibration curve; (a) 10 to 260 kDa and (b) 10.5 to 175 kDa. Table S1: Plackett-Burman design in 12 runs to 6 factors. The response is proteolytic activity. Table S2: Taguchi design. Mixed level design. L18 (6^1 3^3). Table S3: Analysis of Variance for SN ratios using proteolytic activity as the response. Table S4: Results of varying stirrer speed and aeration rate for determining the K_L_a value.
