# Supplementary material for: Scale-Up of the Fermentation Process for the Production and Purification of Serratiopeptidase Using Silkworm Pupae as a Substrate
Source: Methods Protoc. 2024 Feb 25;7(2):19. doi: 10.3390/mps7020019 (PMC10961818; doi:10.3390/mps7020019)
Supplement: Supplementary file 1 [file mps-07-00019-s001.zip › Table S1.pdf]

Table S1. Plackett-Burman design in 12 runs to 6 factors. The response is proteolytic activity.

Trial number	Run order	Silkworm pupae (%)*	Casein (%)*	Soy oil (%)*	$(NH_4)_2HPO_4$ (%)*	$ZnCl_2$ (%)*	$CaCl_2{\cdot}2H_2O$ (%)*	Proteolytic activity (U/mL)**
**9**	1	0.00	0.10	0.10	2.00	0.20	0.20	122.22 ± 31.43
**12**	2	0.00	0.10	0.10	0.50	0.01	0.01	1161.11 ± 86.42
**4**	3	2.50	0.10	2.00	2.00	0.01	0.20	2127.78 ± 7.86
**11**	4	0.00	2.50	0.10	0.50	0.01	0.20	1538.89 ± 7.85
**2**	5	2.50	2.50	0.10	2.00	0.01	0.01	1116.67 ± 86.42
**5**	6	2.50	2.50	0.10	2.00	0.20	0.01	955.56 ± 78.57
**1**	7	2.50	0.10	2.00	0.50	0.01	0.01	2322.22 ± 57.74
**6**	8	2.50	2.50	2.00	0.50	0.20	0.20	4811.11 ± 491.03
**3**	9	0.00	2.50	2.00	0.50	0.20	0.01	366.67 ± 130.99
**8**	10	0.00	0.10	2.00	2.00	0.20	0.01	418.51 ± 35.72
**7**	11	0.00	2.50	2.00	2.00	0.01	0.20	2285.19 ± 289.81
**10**	12	2.50	0.10	0.10	0.50	0.20	0.20	3588.89 ± 172.49

* All percentages refer to the ratio of mass to volume.

** Proteolytic activity is expressed as mean ± SD.
