# Supplementary material for: Scale-Up of the Fermentation Process for the Production and Purification of Serratiopeptidase Using Silkworm Pupae as a Substrate
Source: Methods Protoc. 2024 Feb 25;7(2):19. doi: 10.3390/mps7020019 (PMC10961818; doi:10.3390/mps7020019)
Supplement: Supplementary file 1 [file mps-07-00019-s001.zip › Table S2.pdf]

Table S2. Taguchi design. Mixed level design. L18 (6^1 3^3).

Trial number*	pH	Temperature (°C)	Time (h)	Silkworm pupae ($\%^{w/v}$)	Proteolytic activity (U/mL)
**1**	6	20	24	0.1	874.07 ± 6.42
**2**	7	25	36	0.1	2000.00 ± 245.70
**3**	8	30	48	0.1	644.44 ± 105.99
**4**	6	25	24	0.5	2474.07 ± 99.59
**5**	7	30	36	0.5	3474.07 ± 138.93
**6**	8	20	48	0.5	3477.78 ± 101.84
**7**	6	20	36	1.0	3059.26 ± 222.59
**8**	7	25	48	1.0	3792.59 ± 228.88
**9**	8	30	24	1.0	2700.00 ± 50.92
**10**	6	30	48	1.5	3222.22 ± 189.98
**11**	7	20	24	1.5	3425.93 ± 209.74
**12**	8	25	36	1.5	4900.00 ± 196.58
**13**	6	30	36	2.0	3318.52 ± 241.48
**14**	7	20	48	2.0	4088.89 ± 534.26
**15**	8	25	24	2.0	3874.07 ± 324,39
**16**	6	25	48	2.5	5027.78 ± 296.66
**17**	7	30	24	2.5	2292.59 ± 128.78
**18**	8	20	36	2.5	4262.96 ± 262.55

*The experiment runs were carried out in a random order.
