# Supplementary material for: Scale-Up of the Fermentation Process for the Production and Purification of Serratiopeptidase Using Silkworm Pupae as a Substrate
Source: Methods Protoc. 2024 Feb 25;7(2):19. doi: 10.3390/mps7020019 (PMC10961818; doi:10.3390/mps7020019)
Supplement: Supplementary file 1 [file mps-07-00019-s001.zip › Table S3.pdf]

Table S3. Analysis of Variance for SN ratios using proteolytic activity as the response.

Source	DF	Seq SS	Adj SS	Adj MS	F	P
Silkworm pupae	5	282,998	282,998	56,600	13,41	0,003
pH	2	4,832	4,832	2,416	0,57	0,592
Time	2	28,213	28,213	14,107	3,34	0,106
Temperature	2	39,093	39,093	19,547	4,63	0,061
Residual Error	6	25,329	25,329	4,221		
Total	17	380,466				
