# Supplementary material for: Scale-Up of the Fermentation Process for the Production and Purification of Serratiopeptidase Using Silkworm Pupae as a Substrate
Source: Methods Protoc. 2024 Feb 25;7(2):19. doi: 10.3390/mps7020019 (PMC10961818; doi:10.3390/mps7020019)
Supplement: Supplementary file 1 [file mps-07-00019-s001.zip › Table S4.pdf]

Table S4. Results of varying stirrer speed and aeration rate for determining the K_La value.

Trial number	Stirrer speed (rpm)	volume of air per volume of medium per minute (vvm)	Aeration rate (SL/h)	K_La value (h-1)
1	300	1.5	240	30.47 ± 3.39
2	200	1.5	240	19.41 ± 3.13
3	100	1.5	240	10.69 ± 1.91
4	300	1.0	160	28.36 ± 3.40
5	200	1.0	160	16.05 ± 3.67
6	100	1.0	160	8.68 ± 1.97
7	300	0.5	80	25.45 ± 3.12
8	200	0.5	80	11.05 ± 1.76
9	100	0.5	80	5.94 ± 0.75
