# Supplementary figures and images for: Scale-Up of the Fermentation Process for the Production and Purification of Serratiopeptidase Using Silkworm Pupae as a Substrate
Source: Methods Protoc. 2024 Feb 25;7(2):19. doi: 10.3390/mps7020019 (PMC10961818; doi:10.3390/mps7020019)

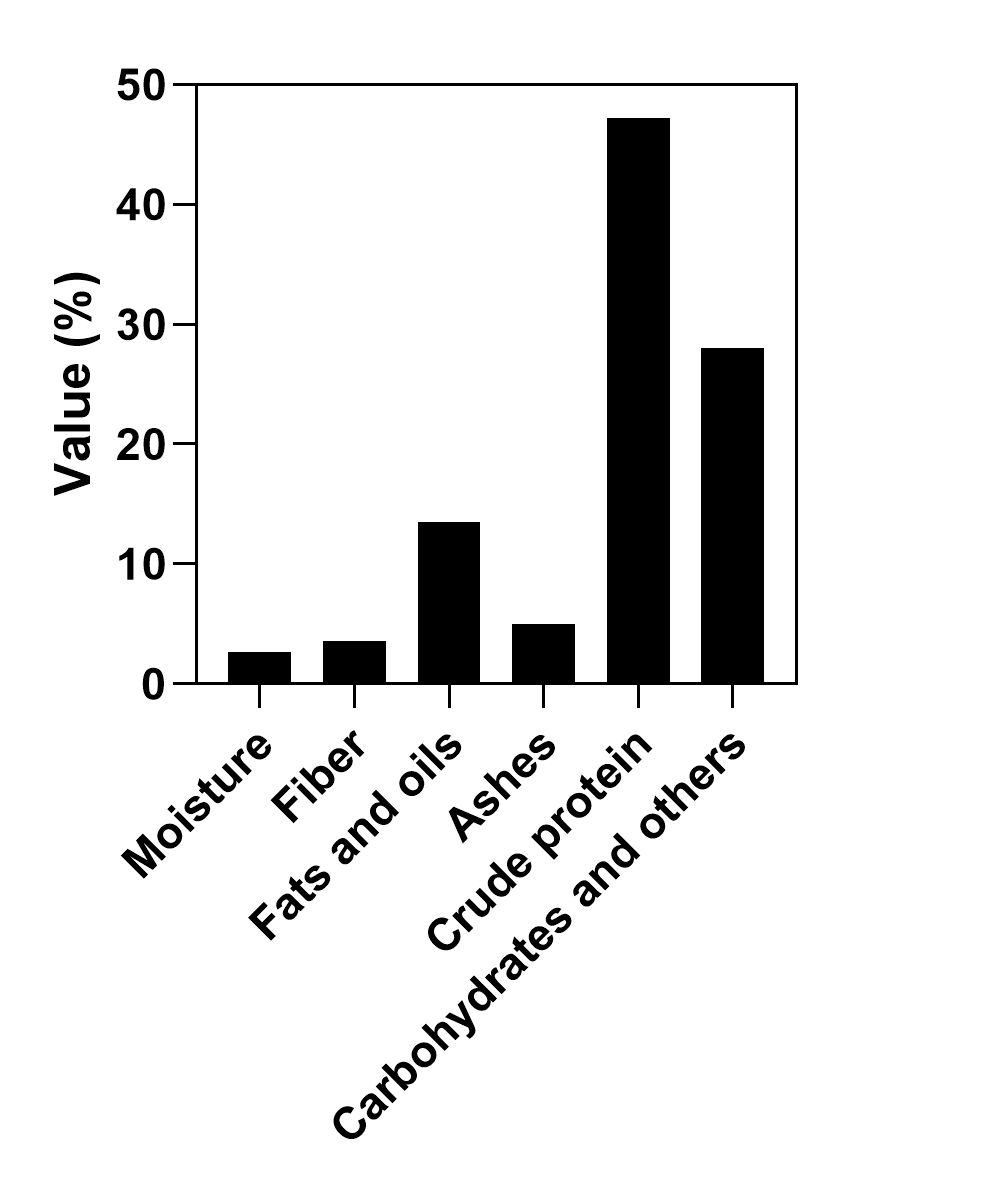

Supplement: Supplementary file 1 [file mps-07-00019-s001.zip › Figure S1.tif]

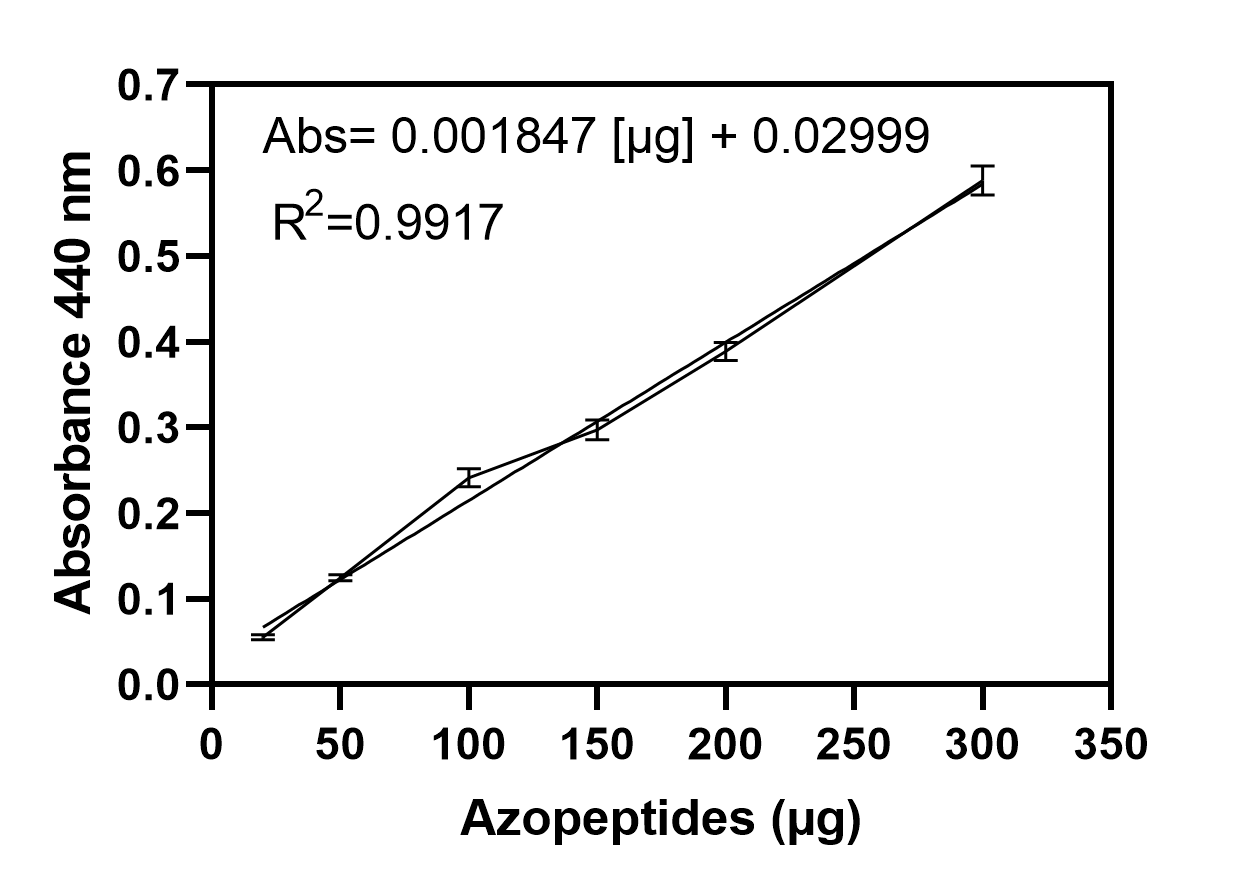

Supplement: Supplementary file 1 [file mps-07-00019-s001.zip › Figure S2.tif]

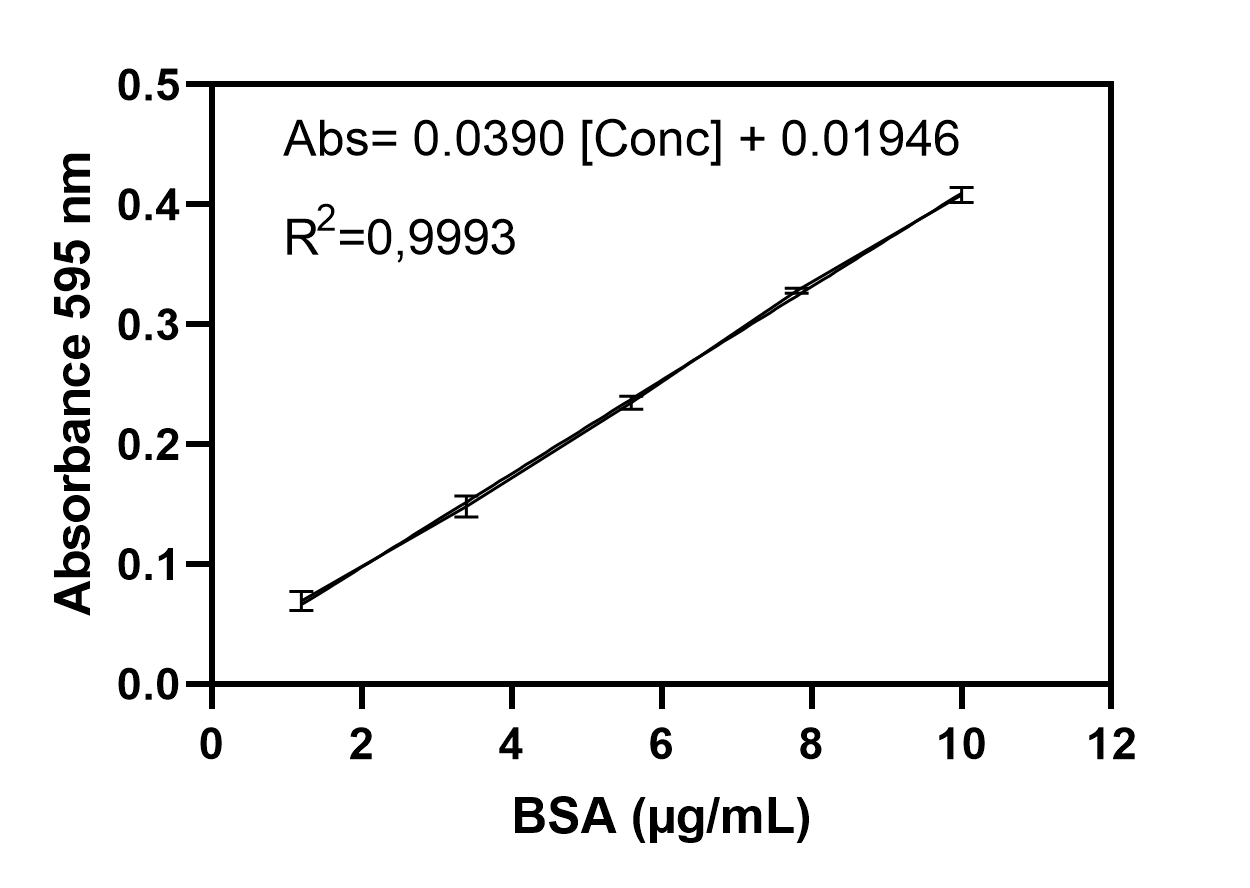

Supplement: Supplementary file 1 [file mps-07-00019-s001.zip › Figure S3.tif]

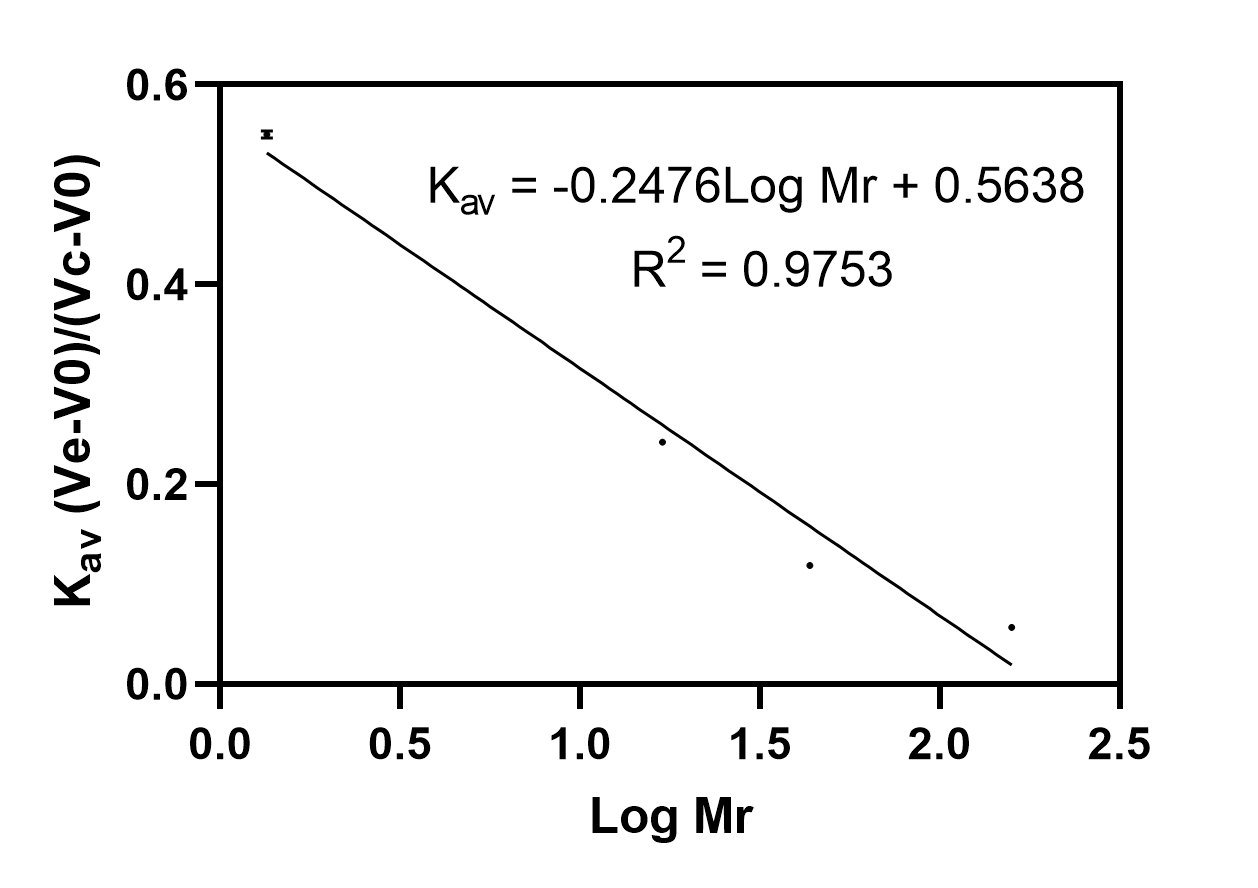

Supplement: Supplementary file 1 [file mps-07-00019-s001.zip › Figure S5.tif]

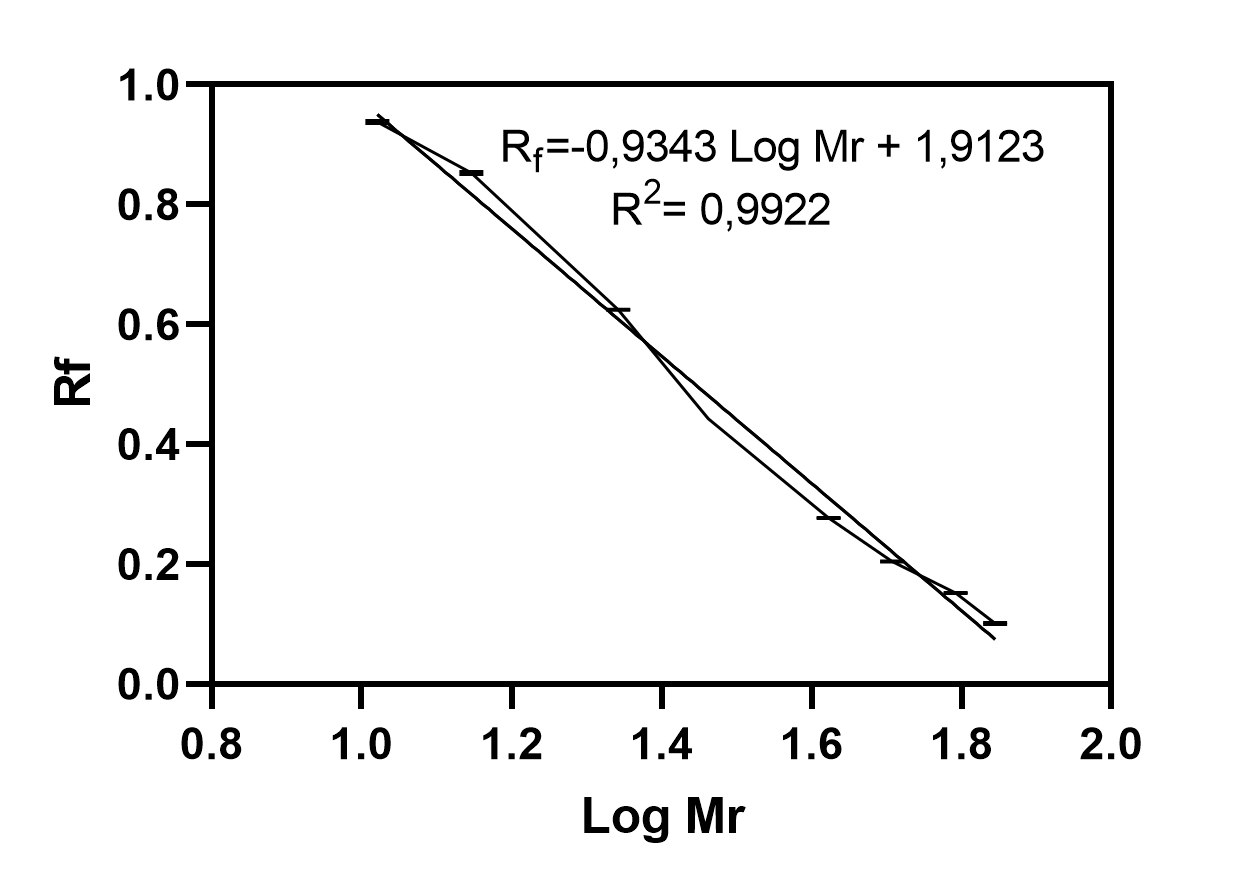

Supplement: Supplementary file 1 [file mps-07-00019-s001.zip › Figure S6 (a).tif]

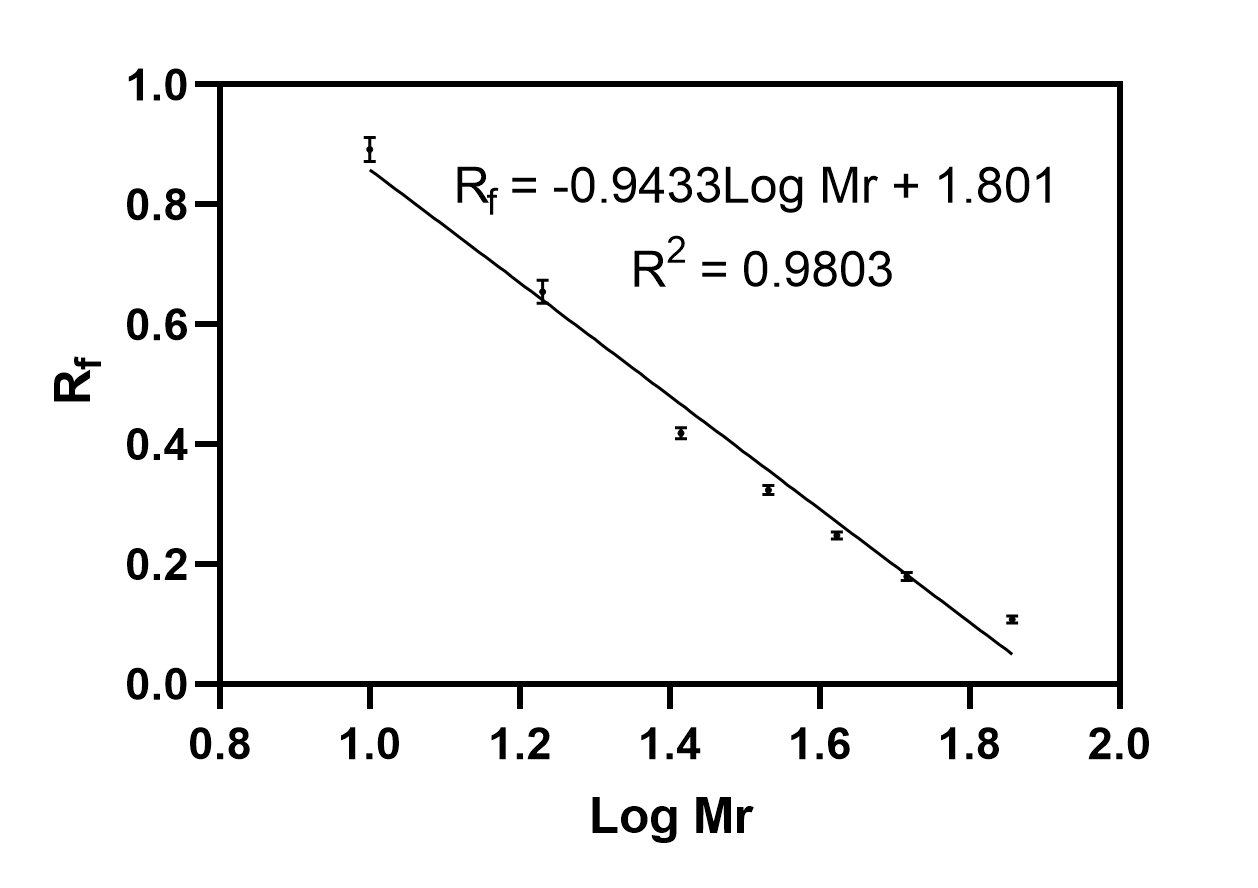

Supplement: Supplementary file 1 [file mps-07-00019-s001.zip › Figure S6 (b).tif]
